# Ketone monoester ingestion preserves working memory during loaded exercise in the heat

**DOI:** 10.1080/15502783.2025.2550199

**Published:** 2025-08-28

**Authors:** Blaine S. Lints, Jenica N. Earl, Riccardo F. Romersi, Maria G. Lombardi, Katelynn T. Persaud, Noah K. Nakagawa, Chimaobim E. Martin-Diala, Gianna F. Mastrofini, Adam T. Harrison, Sten O. Stray-Gundersen, James E. Stampley, Shawn M. Arent, FISSN

**Affiliations:** University of South Carolina, Department of Exercise Science, Arnold School of Public Health, Columbia, SC, USA

**Keywords:** Supplementation, hyperthermia, cognition, task performance

## Abstract

**Background:**

Special Operations Forces (SOF) often operate in extreme heat, where sustained physical exertion may degrade cognition. Exercise-heat stress can reduce cerebral blood flow (CBF) and oxygenation, increasing the risk of catastrophic errors. While ketone monoesters, such as beta-hydroxybutyrate (BHB), may offer neuroprotective effects by improving CBF and energy metabolism, they have yet to be investigated in the context of hyperthermia. This study compared the effects of BHB to carbohydrate (CHO) on cognitive task performance during exercise-heat stress in endurance-trained individuals.

**Methods:**

Using a randomized, double-blind, counterbalanced design, 17 male endurance-trained participants (age = 23.8 ± 5.y; VO_2max_ = 58.6 ± 3.2 ml/kg/min) completed two experimental trials. Exercise was performed in a heat chamber (34°C, 45%RH) and included two 45-min treadmill bouts on a 5% grade at 50% velocity at VO_2max_ while wearing a vest (20% body mass [BM]). Participants consumed 4 mg/kg BM of caffeine at the start of each experimental visit. Before each 45-min bout of exercise, participants ingested either 25 g BHB or 25 g CHO (Cluster Dextrin^TM^). Cognitive performance was assessed pre-, mid-, and post-exercise using 60-s reaction time (RT), Object Hit-and-Avoid (OHA), 1-back, and 2-back tests. Change scores were calculated from baseline and were analyzed using repeated measures ANOVA. Holm-corrected post-hoc analyses were used for significant interactions or main effects (α = 0.05). Effect sizes were calculated as Cohen’s d.

**Results:**

RT was unchanged across time and conditions. OHA accuracy increased over time (*p* < 0.01), but did not differ between conditions. Post hoc testing revealed that accuracy increased from pre- to mid-exercise (+2.4%, *p* = 0.01, d = 0.7), and from pre- to post-exercise (+2.6%, *p* = 0.01, d = 0.8). In the 1-back, a main effect of condition was observed for target accuracy (BHB > CHO +12.6%, *p* = 0.04, d = 0.9), with a significant interaction indicating greater improvements at post-exercise for BHB versus CHO in both target (+23.4%, *p* = 0.03, d = 0.9) and total accuracy (+3.3%, *p* = 0.03, d = 0.7). In contrast, target accuracy declined in CHO at post-exercise (−12.3%, *p* = 0.03, d = 1.0). For the 2-back, the BHB condition resulted in higher total accuracy (+2.2%, *p* = 0.03, d = 0.8) and target accuracy (+13.8%, *p* = 0.02, d = 0.3) compared to CHO.

**Conclusions:**

BHB ingestion may benefit working memory during exercise-heat stress, as evidenced by performance on the 1-back and 2-back. However, no differences were observed in RT or OHA performance, suggesting the benefits of BHB supplementation may be task-specific and most evident during more complex cognitive tasks. These findings indicate that BHB may selectively support some relevant cognitive domains in extreme environments, such as those faced by SOF.

